# Genetic Stability of the Endangered Species *Salix lapponum* L. Regenerated In Vitro during the Reintroduction Process

**DOI:** 10.3390/biology9110378

**Published:** 2020-11-05

**Authors:** Marzena Parzymies, Magdalena Pogorzelec, Katarzyna Głębocka, Elwira Śliwińska

**Affiliations:** 1 Subdepartment of Ornamental Plants and Dendrology, Institute of Horticultural Production, University of Life Sciences in Lublin, 20-950 Lublin, Poland; marzena.parzymies@up.lublin.pl; 2 Department of Hydrobiology and Protection of Ecosystems, University of Life Sciences in Lublin, 20-950 Lublin, Poland; 3 Institute of Plant Genetics, Breeding and Biotechnology, University of Life Sciences in Lublin, 20-950 Lublin, Poland; katarzyna.glebocka@up.lublin.pl; 4 Laboratory of Molecular Biology and Cytometry, UTP University of Science and Technology, 85-789 Bydgoszcz, Poland; elwira@utp.edu.pl

**Keywords:** downy willow, active conservation, micropropagation, ISSR, flow cytometry, somaclonal variation

## Abstract

**Simple Summary:**

*Salix lapponum*, a downy willow, is a boreal relict species, threatened with extinction in Poland. In order to save populations in their refugia on the southern limit of the specie’s range, some activities were undertaken to rebuild their resources. The in vitro propagation was chosen to produce new plants, as it allows obtaining many individuals in a relatively short time with no harm to natural populations. The collected shoot pieces were multiplicated on a special growing media, containing all the necessary macro- and micronutrients, with addition of plant growth regulators to make them form shoots and roots. The obtained plants were then planted into soil and acclimated to natural habitat conditions. On the basis of the conducted genetic analysis and flow cytometry, it was stated that the new plants were genetically unchanged in comparison to the mother plants. The research results confirmed that the tissue culture may be applied in the propagation of the endangered willow species and the obtained plants may be used to build new populations or to strengthen the present ones.

**Abstract:**

*Salix lapponum* L. is a boreal relict species, threatened with extinction in Poland. An 80% decrease in the number of its stands was confirmed in the last half-century, so that to prevent the loss of downy willow, attempts were made to reintroduce this species in natural habitats. Micropropagation was chosen as a first stage of its active conservation. *S. lapponum* shoots were collected and disinfected with NaOCl, AgNO_3_, or HgCl_2_ or with a two-step disinfection with NaOCl and then placed on MS medium with BA 1 mg·dm^−3^ and IBA 0.1 mg·dm^−3^. Regenerated shoots were cultivated with addition of BA, KIN, or 2iP, alone or in combination with auxins, to find the highest multiplication rate. Inter-simple sequence repeat (ISSR) analysis and flow cytometric analyses were conducted on in vitro regenerated plants to check their genetic stability. Disinfection was quite difficult and the use of HgCl_2_ was the most efficient. The highest multiplication rate was obtained in presence of KIN at 0.5 mg·dm^−3^ + IAA at 0.5 mg·dm^−3^. The analysis confirmed the genome size stability, which is in agreement with the results obtained by ISSR, revealing no somaclonal variation in plantlets and therefore allowing the use of the obtained plants for reintroduction.

## 1. Introduction

The genus *Salix* (Salicaceae) comprises over 400 species and has about 65–70 representatives in Europe alone. *Salix* species are woody, heliophilous, pioneer plants, which are able to colonize new habitats. Willows grow in river valleys and wetlands, forming riparian forests or dense shrubs worldwide, from the Arctic to the tropical zone [1]. The genus *Salix* is also of biogeographic importance, because most high mountain willow species in Central Europe are relic taxa with fragmented distribution [2,3,4]. Populations of various species of this genus are spatially limited, and many are present only as female-biased sex ratio populations [5]. 

*Salix lapponum* L. (downy willow), one of the subarctic willows, is a small shrub, growing up to 1–2 m high. It is a dioecious plant, primarily entomophilous and partially anemophilous [6]. It grows separately or in small clusters in lowland and submontane transitional peat bogs, in areas with significant hydration and a small share of woody plants and shrubs in the phytocoenoses. The downy willow prefers acidic peat soil [7]. 

*Salix lapponum* is commonly found in the subarctic and boreal peatlands of northern and northeastern Europe and western Siberia. Isolated populations are present in some mountainous areas of Central and Southern Europe and in Scotland. Refugees of this species have also been confirmed in Poland, at the southern limit of its geographical range. Currently known stands of *S. lapponum* populations are primarily located on the eastern side of the River Vistula and have recently been confirmed to be present in Poleski National Park and Knyszyńska Forest. In Poland, the downy willow is a boreal relict species, considered rare and threatened with extinction [8]. *S. lapponum* has the status of a critically endangered species in the Polish Red Book of Plants. It is also included on the Red list of plants and fungi in Poland. The International Union for Conservation of Nature (IUCN) places it in the EN category (endangered—very high risk of extinction) [7].

Research conducted over the last ten years has shown a decrease in the number of individuals in all populations of *S. lapponum* functioning in eastern Poland. The data show an 80% decrease in the number of stands, as only five of the 35 stands known in the 1950s were confirmed in the last ten years [8]. During the research, downy willow proved able to propagate sexually, as evidenced by the high viability and germinability of its pollen, as well as the high germination capacity of its seeds. The results of genetic studies have also confirmed the absence of clones in populations of this species. However, there were no seedlings observed within the existing populations, most likely due to the unstable habitat conditions, in which seeds were unable to germinate [9].

To prevent the complete extinction of the species in eastern Poland, measures have been undertaken to introduce this species into more advantageous stands or support its existing populations. 

Active conservation includes reintroduction of plants by replanting from other areas or planting newly propagated specimens. As the number of donor plants is usually limited, propagation via tissue culture offers a good alternative, making it possible to produce large numbers of plants in a relatively short time. Furthermore, the plants produced are similar, healthy, and of good quality [10]. It is especially useful when plant taxa cannot be propagated by conventional methods [11].

Tissue cultures have been widely used for propagation of various rare and endangered plant species [12,13,14]. The method has been also applied to propagate various *Salix* species [15,16,17]; however, only one study has been undertaken on micropropagation of *S. lapponum*, and it concerned specimens growing in a mountainous area [4]. 

Somaclonal variation is common in plant tissue cultures, especially in those maintained for prolonged periods of time, when large quantities of exogenous plant growth regulators are used and/or plants are derived from a callus culture [18,19,20,21,22,23]. Therefore, confirmation of the true-to-typeness of micropropagated plants is always recommended and required before reintroduction into natural habitats [24,25,26,27,28]. 

Molecular markers are a very valuable tool for detecting variation in closely related genomes, which may occur between source plants and somaclones regenerated in tissue culture. Molecular marker systems, such as random amplified polymorphic DNA (RAPD) [29], inter-simple sequence repeat (ISSR) [30], or amplified fragment length polymorphism (AFLP) [31], which can be used to estimate somaclonal variation in tissue culture, have both advantages and limitations [26,28,32,33]. There are numerous examples of markers application for this purpose, for example in case of *Robinia ambigua* [34], *Hydrangea macrophylla* [35], *Albizia lebbeck* [36], *Eriophorum vaginatum* [33], *Robinia pseudoacacia* [37], or *Viola uliginosa* [10]. Flow cytometry for somaclone genome size assessment may be used to complement molecular marker analyses [10,25,32,33]. 

In this study, we present a method of in vitro micropropagation for conservation of the endangered *S. lapponum* species. The method was applied as the number of donor plants is low and the trials conducted on generative propagation via seeds or vegetative propagation via cuttings were not satisfactory. Therefore, we aimed to develop a protocol for rapid tissue culture propagation and to examine somaclonal variation using ISSR molecular markers and flow cytometry in order to establish a means of plant propagation that can be applied to large-scale propagule production. 

## 2. Materials and Methods

### 2.1. Tissue Culture Initiation and Stabilization

Pieces of *S. lapponum* shoots were collected from the largest population in eastern Poland, located in a peat bog by Lake Bikcze (51°22′724″ N 023°02′563″ E), at the end of April 2018. The population has a satisfactory sex ratio and high genetic variation [9], which indicates its ability to reproduce sexually. Shoot fragments 5 cm in length were collected from mother plants, which had been previously numbered and whose sex had been verified. Fragments obtained from different specimens were collected separately. The shoot fragments of *S. lapponum* were collected in compliance with a permit issued by the Regional Director for Environmental Protection in Lublin (Permit no. WPN.6400.1.4.2014.JR).

The explants were surface-disinfected in the following steps; washing three times with tap water with detergent; soaking in the mold fungicide Amistar 250 SC (azoxystrobin 25 g·dm^−3^) (Syngenta, Basel, Switzerland), at a concentration of 2 mL dm^−3^; dipping in 70% alcohol for 10 s; and proper disinfection with NaOCl (Chempur, Piekary Śląskie, Poland), at a concentration of 0.5% (10% active chlorine) for 20 min, AgNO_3_ (Sigma-Aldrich, Saint Louis, MO, USA) (0.5%) for 15 min, or HgCl_2_ (Sigma-Aldrich Saint Louis, MO, USA) (0.1%) for 10 s. For the final step, the explants were rinsed three times in sterile distilled water. A two-step disinfection was conducted as well, as described by Skálová et al. [4]. First, the shoot fragments were rinsed in 70% ethanol for 1 min, then soaked in 0.5% NaOCl for 20 min, and then rinsed in sterile distilled water and placed on MS medium. In the second step, after 24 h the same explants were disinfected for the second time in 0.5% NaOCl for 5 min and then rinsed in sterile distilled water. 

The disinfected explants were placed on growing medium prepared according to Murashige and Skoog [38] and supplemented with growth regulators: BA (benzyladenine) (Sigma-Aldrich Saint Louis, MO, USA) at 1 mg·dm^−3^ and IBA (indolebutyric acid) (Sigma-Aldrich Saint Louis, MO, USA) at 0.1 mg·dm^−3^. The pH of the medium was fixed at 5.5 using 1N NaOH or 1N HCl. The medium was solidified with Microbiological Lab-Agar (BioMaxima S.A., Lublin, Poland), at a concentration of 6.75 g·dm^−3^ prior to autoclaving for 21 min at 121 °C and 1 hPa.

The disinfected shoots were cut into single or two-nodal pieces about 2 cm in length and placed individually in tubes. Then they were kept in a growing room at 22 °C during the day and 20 °C at night, with a 16-h photoperiod and a light intensity of 35 µmol·m^−2^·s^−1^. 

All regenerated shoots that were free from contamination were used for further multiplication by subculturing at 6-week intervals on the same medium. After three subcultures, when all shoots with symptoms of contamination had been removed, the shoots were transferred into 500 mL jars for further cultivation. At 6-week intervals they were cut into one- or two-nodal fragments, 2 cm in length and tips separately and placed on the same medium. The process was repeated till enough shoots were obtained to set up the experiments. 

### 2.2. Multiplication

The influence of the growth regulators on the multiplication rate of the *S. lapponum* shoots in the tissue culture was determined. Two-nodal shoot fragments about 2 cm long, with or without an apex, were obtained from a stabilized culture and placed on MS medium supplemented with a growth regulators, i.e., BA (benzyladenine), KIN (kinetin) (Sigma-Aldrich Saint Louis, MO, USA), or 2iP (2-isopentenyl-adenine) (Sigma-Aldrich Saint Louis, MO, USA), at concentrations of 0.5, 1, or 2.5 mg·dm^−3^, respectively, alone or in combination with auxins, i.e., IBA (indole-3-butyric acid) for BA and 2iP or IAA (indole-3-acetic acid) (Aldrich Saint Louis, MO, USA) for KIN, at concentrations 10 times lower.

The cultures were maintained in the same conditions as during the initiation stage. The experiment lasted for five weeks. 

The multiplication rate was calculated as a number of all the explants obtained from the cultivated parts which could be used for further multiplication. 

### 2.3. Rooting and Acclimatization

Rooted plantlets about 7 cm long were removed from the tissue culture and washed under a tap to remove agar residues. The roots were shortened to 2 cm and planted in 1 dm^3^ boxes containing a mixture of acidic peat (pH 3.5–4.5), deacidified peat (pH 5.5–6.5), washed river sand, and perlite in equal volume proportions. Ten plants were planted at random in each box. The boxes were placed in a growing room in fish tanks, which were covered tightly with plastic foil to maintain high humidity. Plants were cultivated in the same conditions as during in vitro cultivation. After two weeks, the foil was gradually removed to harden the plants. After four weeks, plants were transferred individually to P9 (9 × 9 × 9 cm) pots, and a month later, when they had reached a height of about 25 cm, they were cut to about 10 cm to stimulate lateral shoot production. In spring of the following year, after the 15th of May, the plants were ready to be planted in natural habitat.

### 2.4. ISSR Analysis

Plants which were used for genetic analyses were all cultivated in vitro on the MS medium supplemented with KIN 0.5 mg·dm^3^ and IAA 0.05 mg·dm^−3^ to make sure that there were no factors which might have influenced the analysis. We collected 2–3 leaves from each plant. DNA was isolated according to Porebsky et al. [39], with minor modifications. Then, the quality and quantity of DNA was assessed using a Nanodrop ND-1000 spectrophotometer (Thermo Fisher Scientific, Waltham, MA, USA). From a pool of 35 ISSR primers used for screening, nine primers which amplified clear bands were chosen for further analysis (Table 1). Each probe was prepared in a 10 µL mixture containing 1 × PCR buffer (750 mM Tris pH 8.8; 200 mM (NH_4_)_2_SO_4_; 0.1% Tween 20) (Thermo Fisher Scientific, Waltham, MA, USA ) 200 µM of each dNTP, 670 nM of primer, 2 mM MgCl_2_, 40 ng DNA template, and 0.5 U Taq DNA Polymerase (Thermo Fisher Scientific Waltham, MA, USA). Amplification reactions were conducted in a Biometra^®^ (Analytik Jena, Jena, Germany) T1 thermocycler and consisted of 38 cycles with three steps: DNA denaturation for 30 s at 95 °C, primer annealing for 45 s with temperature decreased from 54 °C in the three initial cycles to 53 °C in the next three to 52 °C in the next 32 cycles, and primer extension for 2 min at 72 °C. Before the cycles were begun, initial DNA denaturation was carried for 2 min at 94 °C, and at the end of PCR there was a final extension for 7 min at 72 °C. Products were separated on 2% agarose gels with 0.01% ethidium bromide. 

### 2.5. Flow Cytometry

Flow cytometric analyses were performed on leaves of in vitro regenerated plants. Samples were prepared as previously described [24] using Galbraith’s nuclear isolation buffer [40] supplemented with 2% (w/v) polyvinylpyrrolidone (PVP-10), propidium iodide (PI; 50 μg/mL), and ribonuclease A (50 μg/mL). *Solanum lycopersicum* cv. Stupicke (2C = 1.96 pg/2C) [41] was used as an internal standard. For each sample, PI fluorescence was measured in at least 5000 nuclei, using a CyFlow Ploidy Analyzer flow cytometer (Sysmex Partec, Kobe, Japan). Histograms were analyzed using CyView 1.6 software. The coefficient of variation (CV) of the G0/G1 peak of *Salix* ranged from 3.16% to 6.68%. Nuclear DNA content was calculated based on the linear relationship between the ratios of the 2C peak positions of *Salix*/*Solanum* on a histogram of fluorescence intensities. Genome size was estimated for five control plants (leaves collected in the natural habitat) and 350 in vitro-derived plants.

### 2.6. Statistical Analysis

Statistical analysis of the data was conducted by analysis of variance for orthogonal and non-orthogonal single-factorial design using STATISTICA software. The significance of differences between the means was determined with Tukey’s test at the significance level of *p* = 0.05.

GenAlex 6.502 software was used to determine the proportion of polymorphic bands in each group of clones [42,43].

## 3. Results and Discussion

### 3.1. Tissue Culture Initiation and Stabilization 

Disinfection of shoot fragments was quite difficult. Based on both the number of explants free of contamination and the regeneration rate, the use of mercuric chloride was found to be the most efficient method (Table 2).

According to Mashkina et al. [44], obtaining aseptic cultures of ligneous plants such as willows, with high morphogenetic activity, is one of the most complex stages of in vitro cultures due to the high degree of bacterial or fungal infections. What is more, the regenerative ability of material subjected to disinfection is often reduced or lost. In the present study, irrespective of the disinfection method, the number of explants free of contamination was quite low (maximum 35%). The high level of contamination may have been due to the conditions in which *S. lapponum* is present. The population used as a donor site for explants grows in a peat bog with a large amount of water and high humidity, which enhances the development of fungi. A similar effect was observed by Skálová et al. [4], who, due to a high load of pathogens, used two steps to sterilize shoot explants. However, that method was not successful in the present study. The best results were obtained when the explants were disinfected with mercuric chloride, which has previously been used for other woody plant species [45,46,47].

### 3.2. Multiplication

**Figure 1:**
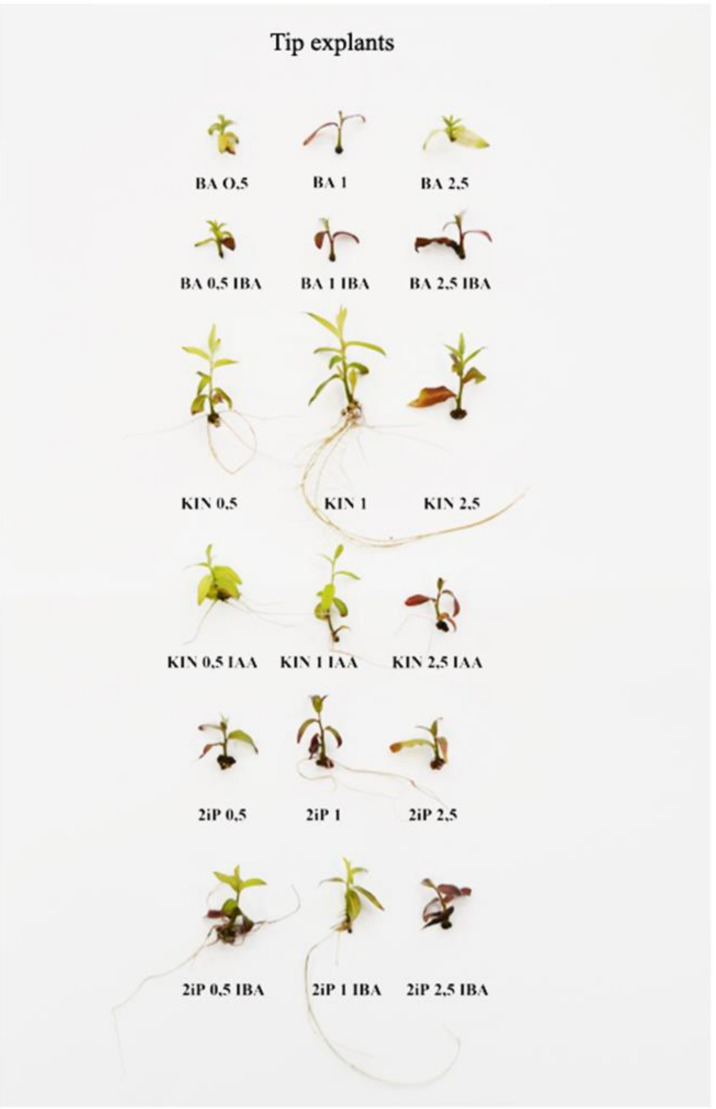
The representative plants obtained from *S. lapponum* shoot tip explants during in vitro cultivation on the media supplemented with different growth regulators.

**Figure 2:**
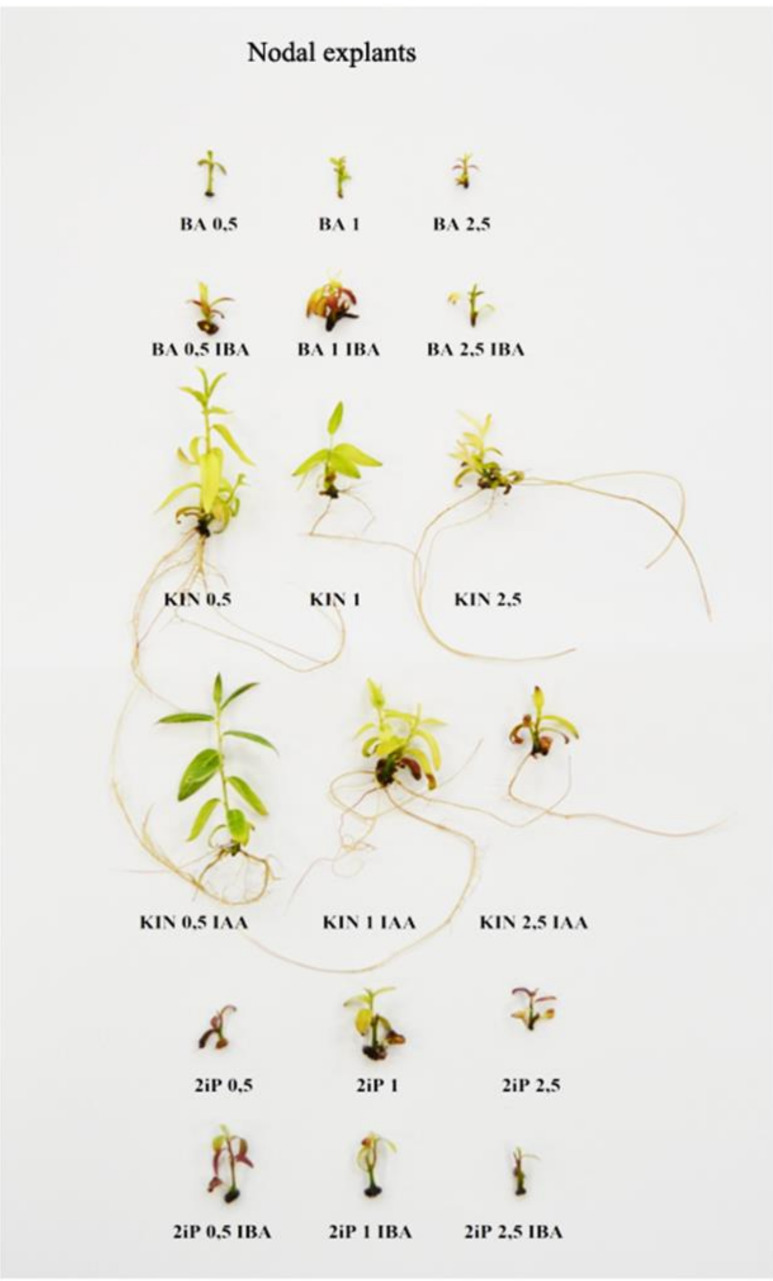
The representative plants obtained from *S. lapponum* shoot nodal explants during in vitro cultivation on the media supplemented with different growth regulators.

The composition of the culture media affected the development, multiplication rate, and rooting of shoots (Table 3 and Table 4; Figure 1 and Figure 2). *S. lapponum* cultivated in tissue culture mainly grows upright, and therefore multiplication entails cutting shoots into smaller pieces with one or two nodes. When these are placed on fresh media they undertake further growth and produce axillary shoots, which may be used as secondary explants for further propagation. During micropropagation, both pieces with an apex and nodal ones without an apex are used.

In the case of tip explants, the most nodes were obtained (5.3 nodes) when kinetin was added to the medium at 0.5 mg·dm^−3^ together with IAA (Table 3). Similar results were obtained in the presence of KIN at a concentration of 1 mg·dm^−3^ without an auxin (4.3 nodes). Production of axillary shoots also depended on the growth regulators used. The most explants formed axillary shoots on media containing BA at 0.5 mg·dm^−3^, BA at 0.5 mg·dm^−3^ + IBA at 0.05 mg·dm^−3^, or KIN at 0.5 mg·dm^−3^ + IAA at 0.05 mg·dm^−3^ (55% each). Good results were also obtained when KIN and IAA were used at the other concentrations (45–50%). The fewest explants formed axillary shoots on media supplemented with 2iP with or without auxins (0–15%) or BA at a concentration of 2.5 mg·dm^−3^ (15%). On the other hand, when benzyladenine was used at the highest concentration, explants formed the highest number of shoots (1.5) among all treatments. 

Nodal explants, with the apex removed, produced axillary shoots in all treatments (Table 3). The most branched shoots were noted on the medium supplemented with KIN at 1 mg·dm^−3^ + IAA at 0.1 mg·dm^−3^ (70%). Good results were also obtained in the presence of KIN at 0.5 mg·dm^−3^ (65%), BA at 2.5 mg·dm^−3^ + IBA at 0.25 mg·dm^−3^, KIN at 0.5 mg·dm^−3^ + IAA at 0.05 mg·dm^−3^ (55% each), or KIN at 2.5 mg·dm^−3^ (50%). The number of axillary shoots was the same in all treatments (1.0).

The features described above were used to calculate the multiplication rate (Table 3). In case of tip explants, the highest multiplication rate was obtained when explants were cultivated on the medium supplemented with KIN at 0.5 and IAA at 0.1 mg·dm^−3^ (5.85). Good results were also observed on the media with KIN1 (4.70), BA 0.5 (4.39), KIN 0.5 (4.15), or KIN 1 together with IAA 0.1 mg·dm^−3^ (4.15). Multiplication rate obtained from nodal explants were definitely lower. The highest one was obtained when nodes were placed on the media supplemented with KIN 1 and IAA 0.1 mg·dm^−3^ (0.75). None of the treatments allowed obtaining a multiplication rate above 1.0. 

Taking into consideration that both nodes and tips are used during cultivation of *Salix lapponum* in vitro, the multiplication rate for both types of explants was also counted. The highest multiplication rate was obtained on the medium supplemented with KIN at 0.5 mg·dm^−3^ + IAA at 0.5 mg·dm^−3^ (6.45). Satisfactory multiplication rates were also noted in the presence of BA at 0.5 mg·dm^−3^, KIN at 0.5 and 2.5 mg·dm^−3^, KIN at 1 mg·dm^−3^+ IAA at 0.1 mg·dm^−3^, and 2iP at 1 mg·dm^−3^ (from 4.0).

According to Chalupa [45], willows growing in tissue culture on appropriate culture media are able to grow quickly and produce roots, and plants can be propagated quickly by dividing the stems, which leads to large numbers of node segments. Cytokinins have been shown to be essential during in vitro propagation of plants, but the optimal concentration is different for each species. Cytokinins play an important role in DNA synthesis and cell division, which may stimulate the induction of shoots. The reaction of plants to growth regulators is linked to the concentrations of various types of cytokinins [48]. In the case of willow species, the most commonly used growth regulators are KIN, BA, IAA, IBA, NAA, BA, and GA_3_ [49]. The authors cited used kinetin in different concentrations for multiplication of three varieties of *Salix viminalis* and found that the medium containing 0.5 mg·dm^−^ of kinetin was the most advantageous. Skálová et al. [4] cultivated *S. lapponum* in tissue culture on MS medium supplemented with BA at a concentration of 0.1 mg·dm^−3^ and IBA at 0.01 mg·dm^−3^, and noted a high multiplication rate for shoots on that medium. Brandova et al. [50] also used MS media supplemented with 0.1 mg·dm^−3^ BA and 0.01 mg·dm^−3^ IBA. 

*Salix lapponum* shoots rooted spontaneously in the tissue culture. The percentage of rooted plantlets and the number of roots depended on the growth regulators added to the medium (Table 4).

Tip explants did not produce roots when the medium was supplemented with benzyladenine, alone or in combination with an auxin. There were no rooted plantlets in the presence of the highest concentration of KIN used together with IAA or in the presence of 2iP at a concentration of 2.5 mg·dm^−3^, used alone or in combination with IBA. The most rooted plantlets were noted when KIN was used at 1 mg·dm^−3^ (50%). Fairly good results were also obtained on the medium with 2iP at 0.5 mg·dm^−3^ (45%) or KIN at 0.5 mg·dm^−3^, alone or with IAA at a concentration of 0.05 mg·dm^−3^ (40%). The most shoots per explant were obtained on the medium supplemented with KIN at 0.5 mg·dm^−3^ and IAA at 0.05 mg·dm^−3^ (3.75), and the fewest in the case of 2iP at 1 mg·dm^−3^ (1.25).

In the case of nodal explants, the highest number of rooted plants was obtained on the medium supplemented with KIN at 0.5 mg·dm^−3^ and IAA at 0.05 mg·dm^−3^ (65%). Good results were also obtained in the presence of KIN at 0.5 mg·dm^−3^, KIN at 1 mg·dm^−3^ + IAA at 0.1 mg·dm^−3^, or 2iP at 1 mg·dm^−3^ + IBA 0.1 mg·dm^−3^ (55%). No roots were observed in the presence of BA in any combination, 2iP at concentrations of 0.5–1 mg·dm^−3^, or the highest concentration of 2iP together with IBA. The number of roots per shoot depended on the growth regulators added to the medium. On the medium supplemented with KIN at 0.5 mg·dm^−3^ or KIN at 2.5 mg·dm^−3^ + IAA at 0.25 mg·dm^−3^, there were significantly more roots (3.45 and 3.50, respectively) than in the case of 2iP at a concentration of 0.5 or 1.0 mg·dm^−3^ used together with IBA (1.83 and 1.55, respectively). 

Spontaneous rooting is often observed in willow species cultivated in tissue culture on media containing cytokinins and auxins. Skálová et al. [4] observed significant rooting of *S. lapponum*, while Grendysz et al. [49] observed that kinetin in various concentrations did not significantly affect rooting of *Salix viminalis* in tissue culture. However, the addition of 0.5 mg·dm^−3^ of kinetin was most effective in stimulating the spread of the roots.

The growth and development of tip and nodal explants of *S. lapponum* are presented on Figure 1 and Figure 2. Taking into consideration all the evaluated features of the cultivated shoots it might be stated that the media which allows to obtain a lot of good quality and rooted shoots, both for tip and nodal explants to make the work easier, is the MS medium supplemented with KIN 0.5 mg·dm^−3^ and IAA 0.05 mg·dm^−3^. 

### 3.3. ISSR

From all regenerated plants, 66 were randomly selected for ISSR analysis. They were obtained from four different mother plants and according to their origin divided into four groups described as C1–C4. As mother plants were not available for molecular marker analysis, 11 specimens from the natural environment and from the same population, were used as a reference, hereafter referred to as wild forms.

A total of 111 bands were obtained in the study and they can be divided into three groups (Table 5):

- Nineteen products were unique to wild forms.
- Ten products were amplified in at least one group of regenerated plants but absent in wild forms.
- Eighty-two products were present in wild forms and at least one group of regenerated plants. Seventeen of them were monomorphic in all analyzed individuals.

Polymorphism of regenerated plants ranged from 13.51% to 45.05%. Altogether polymorphic bands constituted 39.10% in all analyses. The size range of all amplified PCR products was 180–3000 bp.

In the vast majority of articles concerning in vitro plant regeneration, the band patterns of the molecular marker are monomorphic [33,35,51,52]. However, there are some reports of polymorphic products of tissue culture-propagated plants [10,34,37]. In the case of downy willow, the share of such bands was quite high, although much smaller than in wild forms (80.18%), which may indicate somaclonal variation. Nevertheless, there were only three products that were private for two clonal forms (two in C2 and one in C1), while the other bands absent in wild forms were present in at least two groups. There are two possible explanations for products amplified only in regenerated plants. First, they are new DNA fragments absent in *S. lapponum*. Taking into account high share of polymorphic products in regenerated plants, some genome rearrangements cannot be excluded. However, if they took place, they were minor changes, as there were no morphological differences between regenerated and wild type plants. Second, DNA fragments unique to regenerated plants in this research are naturally present in downy willow. As this species is highly polymorphic [8,53], 11 individuals may do not reflect its variability. It also needs to be highlighted that there were 17 bands monomorphic in wild forms and they were also monomorphic in regenerated plants. These results suggest that tissue-culture-cultivated *S. lapponum* specimens correspond morphologically to the habit of the species with minor genetic rearrangements that could have appear in rather non-coding regions of the genome.

### 3.4. Flow Cytometry

The nuclear DNA content of *S. lapponum* was 0.874 ± 0.019 pg/2C in the leaves of control plants collected from the natural environment and 0.867 ± 0.021 pg/2C in plantlets produced in vitro. This confirmed the genome size stability of micropropagated material, which is in agreement with the results obtained by ISSR, revealing no somaclonal variation in plantlets. Similar stability has been found for numerous species produced in vitro, including trees and shrubs, e.g., oak, eucalyptus, banana, olive, and Japanese quince [54,55,56,57,58].

To the best of our knowledge, the genome size of *S. lapponum* had not previously been established. However, for various *Salix* species it has been found to vary between 0.70 pg/2C and 1.72 pg/2C [59]. The value established here (about 0.87 pg/2C) falls within this range.

## 4. Conclusions

Research conducted to date suggests that in vitro cultures of endangered species can be a key stage of active conservation, making it possible to preserve the genetic resources of willows and other plants threatened with extinction.

The tissue culture protocol presented here may be applied in the propagation of the endangered willow species which could be then used for introduction into new stands or to strengthen the existing populations. The results of the genetic analyses are also presented, which allow estimating if the in vitro cultivation does not change the genetic structure of the future population or to eliminate unwanted changes.

## Figures and Tables

**Table 1:** Inter-simple sequence repeat (ISSR) primers used for analysis with range of band sizes.

Primer	Sequence	Size Range of Bands
SR16	(GA)_8_C	250–1700
SR22	(CA)_8_G	350–1600
SR32	(AG)_8_YT	180–2200
SR68	(AC)_8_T	300–2600
SR75	(AT)_8_C	750–2000
SR77	(ATG)_8_C	500–1800
SR78	(ATG)_8_G	400–1700
SR84	(CA)_8_RG	510–3000
SR85	(CA)_8_RT	420–1800

Y—T or C; R = G or A

**Table 2:** Disinfection efficiency and regeneration rate of *S. lapponum* explants depending on the disinfection method.

Disinfection Method	Number of Regenerating Explants without Contamination n/N (%)
NaOCl 0.5%	18/118 (21.7)
AgNO_3_ 0.5%	6/132 (4.5)
HgCl_2_ 0.1%	42/120 (35.0)
Two-step disinfection	41/229 (18.0)

n—number of uncontaminated regenerating shoots, N—number of explants used.

**Table 3:** Regeneration and multiplication of *S. lapponum* shoots in tissue culture depending on the growth regulators added to the medium.

Growth Regulators	Tip Explants				Nodes			Tips and Nodes Mn Rate
	Number of Nodes per Explant	Plants with Axillary Shoots (%)	Axillary Shoots/Explant	Mn Rate **	Plants with Axillary Shoots (%)	Axillary Shoots/Explant	Mn Rate	
BA 0.5	3.8_bc *_	55	1.08_b_	4.39	30	1.00_a_	0.30	4.69
BA 1	3.0_b-e_	30	1.00_b_	3.30	25	1.00_a_	0.25	3.55
BA 2.5	2.8_c-e_	15	1.50_a_	3.02	35	1.00_a_	0.35	3.39
BA 0.5 + IBA 0.05	2.7_c-e_	55	1.00_b_	3.25	25	1.00_a_	0.25	3.53
BA 1 + IBA 0.1	2.1_e_	40	1.00_b_	2.5	40	1.00_a_	0.40	2.9
BA 2.5 + IBA 0.25	2.5_de_	30	1.14_b_	2.84	55	1.00_a_	0.55	3.39
KIN 0.5	3.9_bc_	25	1.00_b_	4.15	65	1.00_a_	0.65	4.8
KIN 1	4.3_ab_	40	1.00_b_	4.70	45	1.00_a_	0.45	5.15
KIN 2.5	3.6_b-d_	23	1.00_b_	3.83	50	1.00_a_	0.50	4.33
KIN 0.5 + IAA 0.05	5.3_a_	55	1.00_b_	5.85	55	1.09_a_	0.60	6.45
KIN 1 + IAA 0.1	3.7_b-d_	45	1.00_b_	4.15	70	1.07_a_	0.75	4.9
KIN 2.5 + IAA 0.25	2.8_c-e_	50	1.10_b_	3.35	39	1.00_a_	0.39	3.74
2iP 0.5	3.3_b-e_	0	-	3.30	23	1.00_a_	0.23	3.53
2iP 1	3.5_b-d_	5	1.00_b_	3.55	45	1.00_a_	0.45	4.0
2iP 2.5	2.5_de_	0	-	2.50	40	1.00_a_	0.40	2.5
2iP 0.5 + IBA 0.05	3.3_b-e_	15	1.00_b_	3.45	45	1.00_a_	0.45	3.9
2iP 1 + IBA 0.1	3.3_b-e_	5	1.00_b_	3.35	35	1.00_a_	0.35	3.7
2iP 2.5 + IBA 0.25	2.9_b-e_	15	1.00_b_	3.05	25	1.00_a_	0.25	3.3

* Values in columns with the same letter do not differ significantly at *p* = 0.05. ** Mn rate—multiplication rate

**Table 4:** Rooting of *S. lapponum* shoots in tissue culture depending on the growth regulators added to the medium.

Growth Regulator	Tip Explants		Nodes	
	Rooted Plantlets (%)	Number of Roots per Plantlet	Rooted Plantlets (%)	Number of Roots per Plantlet
BA 0.5	0	-	0	-
BA 1	0	-	0	-
BA 2.5	0	-	0	-
BA 0.5 + IBA 0.05	0	-	0	-
BA 1 + IBA 0.1	0	-	0	-
BA 2.5 + IBA 0.25	0	-	0	-
KIN 0.5	40	2.75_ab_*	55	3.45_a_
KIN 1	50	2.80_ab_	30	2.17_ab_
KIN 2.5	23	2.00_ab_	25	2.20_ab_
KIN 0.5 + IAA 0.05	40	3.75_a_	65	2.62_ab_
KIN 1 + IAA 0.1	35	2.29_ab_	55	2.64_ab_
KIN 2.5 + IAA 0.25	0	-	15	3.50_a_
2iP 0.5	45	2.22_ab_	0	-
2iP 1	20	1.25_b_	0	-
2iP 2.5	0	-	20	2.00_ab_
2iP 0.5 + IBA 0.05	30	2.64_ab_	30	1.83_b_
2iP 1 + IBA 0.1	17	2.14_ab_	55	1.55_b_
2iP 2.5 + IBA 0.25	0	-	0	-

* Values in columns with the same letter do not differ significantly at *p* = 0.05.

**Table 5:** Numbers of ISSR products according to groups of plants.

Types of PCR Products		C1	C2	C3	C4
Unique to wild forms	19	-	-	-	-
Unique to regenerated plants	10	6	7	4	4
Wild forms and regenerated plants	82	76	71	67	66
Polymorphism (%)	-	45.05	13.51	27.93	28.83

## Abbreviations

MDPI	Multidisciplinary Digital Publishing Institute
DOAJ	Directory of open access journals
TLA	Three letter acronym
LD	linear dichroism
MS	Murashige and Skoog medium
BA	benzyladenine
KIN	kinetin
2iP	2-isopentenyl-adenine
IBA	indolebutyric acid
IAA	indole-3-acetic acid
NaOCl	sodium hypochlorite
AgNO_3_	silver nitrate
HgCl_2_	mercuric chloride
ISSR	inter-simple sequence repeat
RAPD	random amplified polymorphic DNA
AFLP	amplified fragment length polymorphism
PCoA	principal coordinate analysis
Mn rate	multiplication rate
